# Cytomegalovirus Meningoencephalitis in a Critically Ill Elderly Female: A Case Report

**DOI:** 10.7759/cureus.22611

**Published:** 2022-02-25

**Authors:** Anna Anjelica R Sanchez, Archimedes Apa-ap, Jennifer Chua

**Affiliations:** 1 Department of Internal Medicine, Section of Neurology, Cardinal Santos Medical Center, San Juan City, PHL; 2 Department of Clinical Neurosciences, Section of Neurology, University of the East Ramon Magsaysay Memorial Medical Center, Quezon City, PHL; 3 Department of Internal Medicine, Cardinal Santos Medical Center, San Juan City, PHL

**Keywords:** viral meningoencephalitis, immunocompetent adult, cmv encephalitis, critically ill elderly patients, cytomegalovirus (cmv)

## Abstract

Cytomegalovirus (CMV) disease is usually a mild and self-limiting disease in immunocompetent patients. Recent evidence shows that CMV infection may also develop in the setting of critical illness, burn and sepsis and is usually associated with increased mortality rate and prolonged ICU stay. This paper describes an 83-year-old female who was initially admitted as a case of community-acquired pneumonia-high risk but remained febrile with paucity of verbal output despite correction of pneumonia and other electrolyte derangements. MRI showed the presence of peculiar-appearing signal abnormalities in the interhemispheric region and the anterior frontal convexities which were suspected to represent secondarily infected fluid collections. On lumbar tap, viral cerebrospinal fluid (CSF) panel showed a positive result for CMV infection. The patient was then given ganciclovir for 14 days followed by valganciclovir for three months. The most notable improvement was noted with the lysis of fever several days after starting anti-viral treatment. Verbal output remained limited, yet, on repeat tap after completion of treatment, CMV viral panel is now negative.

## Introduction

Cytomegalovirus (CMV) infection is commonly seen in immunocompromised patients such as those with AIDS or post-transplant patients. However, in immunocompetent individuals, severe CMV infection is rarely reported. Sepsis and mechanical ventilation show a strong association with the development of CMV infection in these patients [1]. The central nervous system is the second most frequent site of CMV infection in the immunocompetent presenting as meningitis, encephalitis or transverse myelitis [2]. In this paper, a case of an 83-year-old female who developed CMV meningoencephalitis will be discussed.

This paper was presented as an E-poster presentation in the 31st European Congress of Clinical Microbiology & Infectious Diseases (ECCMID) held July 9-12, 2021, in Vienna, Austria.

## Case presentation

The patient is an 83-year-old female who was initially admitted due to fever of seven days duration. She was managed as a case of community-acquired pneumonia - high risk and was referred to Neurology service due to persistently decreased sensorium. Cranial MRI at that time was unremarkable. EEG showed generalized slowing of background activity with frequent bilateral triphasic waves over the bilateral frontal head regions and was consistent with metabolic encephalopathy. She started to improve clinically after antibiotic therapy and correction of other metabolic derangements.

However, on the 10th hospital day, the patient had sudden onset desaturation and was eventually intubated and hooked to a mechanical ventilator. On the 12th hospital day, the patient’s condition improved and she was noted to be awake, responds through gestures with spontaneous and purposeful movement of all extremities. She was finally extubated after three days at the ICU and was transferred to a regular room.

**Figure 1:**
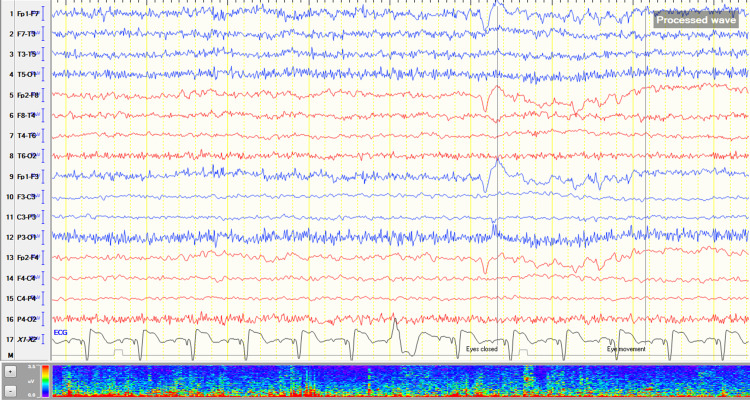
Electroencephalogram on the 22nd hospital day showing mild generalized slowing of background activity.

**Figure 2:**
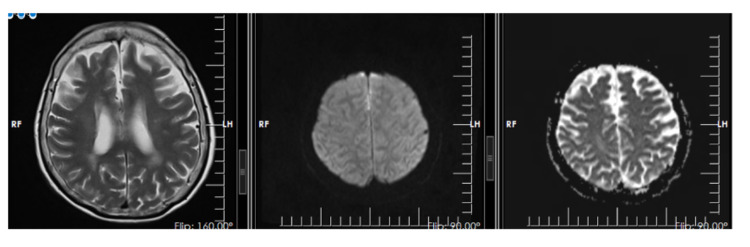
Cranial MRI done on the 22nd hospital day showing very peculiar-appearing signal abnormalities demonstrating restricted diffusion involving the interhemispheric region and the anterior frontal convexities of uncertain etiology, but are suspected to represent secondarily infected fluid collections.

On the 15th hospital day, the patient’s fever recurred and persisted. She was worked up for infectious causes of fever but the work-up was unremarkable. During this time, the patient was noted to have no verbal output and on the 22nd hospital day, was again seen to have increased sleeping time. A repeat EEG was done which showed mild generalized slowing of the background activity with significant and improved response to stimulation (Figure 1). Triphasic waves were no longer seen. A repeat cranial MRI with contrast was done this time showing very peculiar-appearing signal abnormalities demonstrating restricted diffusion involving the interhemispheric region and the anterior frontal convexities of uncertain etiology, but are suspected to represent secondarily infected fluid collections (Figure 2). These fluid collections were not present in the cranial MRI done on the fourth hospital day. Due to these findings and the absence of an obvious source of infection, a lumbar tap was done. Opening and closing pressures were noted to be within normal range, at 100mmH20 and 40mmH20. Cerebrospinal fluid (CSF) was noted to be clear and free flowing. Routine analysis showed the presence of elevated protein at 867mg/L and slightly decreased glucose levels at 47%. CSF cultures, tuberculosis (TB) gene Xpert, Cryptococcal Antigen Latex Agglutination System (CALAS) and India ink were negative. CSF viral panel was positive for CMV with a viral load of 18,400 copies per ml.

Upon diagnosis, the patient was given ganciclovir 250mg IV in 100cc plain normal saline solution (PNSS) every 12 hours. In the interim, the patient had lysis of fever with improved wakefulness but still with minimal verbal output. After completing 14 days of gancyclovir, valganciclovir 450mg/tab two tablets per day was started and was completed for three weeks.

After completing treatment, the patient remained fever-free, however, improvement with regard to verbal output was not as dramatic. She was noted to be awake, with regard, follows some commands, with occasional verbal output, tolerating rehab and now able to sit at bedside chair. Repeat lumbar tap was done and this time CSF CMV viral panel turned out negative.

## Discussion

Cytomegalovirus infection is common with seroprevalence rates increasing progressively from 65% among those aged 40 to 49 years old to 91% in those aged 80 years and above, most notably in immunocompromised individuals [3]. This virus, however, does not usually cause significant morbidity and mortality in immunocompetent patients. In these patients, critical illness, intubation and mechanical ventilation, sepsis and burns are associated with CMV infection [1,4-7]. It has been suggested that CMV infection in immunocompetent patients is a result of reactivation of a latent CMV infection brought about by the aforementioned risk factors [7,8]. However, reinfection from an exogenous strain is also a possibility [8]. In the critically ill, CMV infection is seen in 0-36% of patients and usually occurs between four and 12 days after onset of illness [7]. Gastrointestinal tract abnormalities as well as pneumonitis are commonly seen in these subsets of patients, however, few cases have also shown central nervous system (CNS) involvement [2].

The most common CNS manifestation of CMV infection in immunocompetent patients is acute inflammatory demyelinating polyradiculoneuropathy or Guillain-Barré-Strohl syndrome, wherein CMV represents the cause in 10-22% of the cases [3]. Other rarer neurologic manifestations include myelitis, encephalomyelitis, encephalitis, meningoencephalitis, meningitis, or meningoradiculopathy [2,8]. Two clinical forms of CMV meningoencephalitis have been described. The first form is the paroxysmal type which is characterized by focal neurologic signs and symptoms, alternating side of neurological deficits, and headache. Symptoms may last from minutes to hours, and generally has a benign outcome. The second type is the monophasic type and is characterized by more frequent occurrence of seizures, altered sensorium, symptoms lasting for days which may result in complete or partial recovery or even death [9]. The patient’s clinical presentation and temporal profile appear to be consistent with the monophasic type of meningoencephalitis.

In immunocompromised patients, guidelines regarding treatment of severe CMV infection are in place but this does not hold true for immunocompetent patients There are several case reports and reviews which show rapid clinical improvement and better outcomes after starting anti-CMV therapy, however, these studies concluded that clinical trials are still needed to prove whether these are truly beneficial [5,10,11]. One of the main dilemmas in CMV infection in an immunocompetent patient is the decision whether to treat or not since most cases are self-limited with few sequelae. Another concern is the potential side effects of anti-CMV therapy, especially in the critically ill. Ganciclovir and foscarnet, which are recommended by the Expert Panel of the Infectious Diseases Society of America as drugs of choice for CMV encephalitis in immunocompromised patients, are notorious for causing myelosuppresion and renal toxicity [10,12]. There is a paucity of data regarding the safety and efficacy of these drugs in immunocompetent individuals since these cases are rarely reported [5,11]. In this case, the decision to treat the patient was made due to the severity of the illness based on the clinical status and viral load. No adverse drug reactions were noted during the course of treatment.

In a study by Eddleston et al., 10 patients were identified with isolated CMV encephalitis out of 34 immunocompetent patients with severe CMV infection. In those 10 patients, no one died, however one patient remained disabled and dysphasic. The prognosis in patients with isolated CMV encephalitis appears to be excellent compared to those with multiorgan or non-CNS infection [11]. After treatment, the most glaring evidence of resolution of infection in this patient was the lysis of fever and the non-detection of the virus in the CSF. The patient’s neurologic condition improved slightly, however, the paucity of speech persisted.

## Conclusions

A rare case of a monophasic type of CMV meningoencephalitis in an immunocompetent patient who was initially admitted as a case of sepsis from community-acquired pneumonia was presented. In the review of literature, it was noted that, in addition to an immunocompromised state, there is indeed an association of other clinical conditions like critical illness and sepsis in the development of CMV infection. This infection has a wide array of vague neurologic manifestations including meningoencephalitis which can prove to be diagnostically challenging in a critically ill patient. CNS involvement may be self-limiting but some cases, including the case presented, were shown to have permanent residual disabilities. Clinical guidelines in the management of symptomatic CMV infections in an immunocompetent patient are not well established. Further studies may contribute to addressing the gaps in the diagnosis and management of severe CMV meningoencephalitis in immunocompetent patients as these may have permanent and life-threatening sequelae if not adequately managed. 

## References

[1] Al-Omari A, Aljamaan F, Alhazzani W, Salih S, Arabi Y (2016). Cytomegalovirus infection in immunocompetent critically ill adults: literature review. Ann Intensive Care.

[2] Rafailidis PI, Mourtzoukou EG, Varbobitis IC, Falagas ME (2008). Severe cytomegalovirus infection in apparently immunocompetent patients: a systematic review. Virol J.

[3] Staras SA, Dollard SC, Radford KW, Flanders WD, Pass RF, Cannon MJ (2006). Seroprevalence of cytomegalovirus infection in the United States, 1988-1994. Clin Infect Dis.

[4] Ku YH, Chuang YC, Yu WL (2018). Post Sepsis Cytomegalovirus Syndrome in Critically Ill Patients. https://www.researchgate.net/publication/325594117_Post_Sepsis_Cytomegalovirus_Syndrome_in_Critically_Ill_Patients.

[5] Lancini D, Faddy HM, Flower R, Hogan C (2014). Cytomegalovirus disease in immunocompetent adults. Med J Aust.

[6] Mansfield S, Grießl M, Gutknecht M, Cook CH (2015). Sepsis and cytomegalovirus: foes or conspirators?. Med Microbiol Immunol.

[7] Osawa R, Singh N (2009). Cytomegalovirus infection in critically ill patients: a systematic review. Crit Care.

[8] Micallef S, Galea R (2018). CMV encephalitis in an immune-competent patient. BMJ Case Rep.

[9] Devetag FC, Boscariolo L (2000). Cytomegalovirus meningoencephalitis with paroxysmal course in immunocompetent adults: a new nosographical entity. Clinical, diagnostic and therapeutic correlations, and pathogenetic hypothesis. Eur Neurol.

[10] Nangle S, Mitra S, Roskos S, Havlichek D (2018). Cytomegalovirus infection in immunocompetent adults: is observation still the best strategy?. IDCases.

[11] Eddleston M, Peacock S, Juniper M, Warrell DA (1997). Severe cytomegalovirus infection in immunocompetent patients. Clin Infect Dis.

[12] Tunkel AR, Glaser CA, Bloch KC (2008). The management of encephalitis: clinical practice guidelines by the Infectious Diseases Society of America. Clin Infect Dis.

